# Diagnosis or "Proof."

**Published:** 1898-11-26

**Authors:** 


### DIAGNOSIS OR "PROOF."

The discussion which took place at the last meeting of the Medical Society on a case exhibited by Mr. Battle, in which various tumours, which had been diagnosed as sarcoma, had disappeared under the use of Coley's fluid, brought into considerable prominence the question of diagnosis in such cases. This particular case had already been exhibited at a meeting of the society while under the treatment. He then presented a large tumour under the right clavicle extending down the chest, another above the clavicle extending into the posterior triangle of the neck, and others over the sternum, with numerous diseased glands in the neck. The treatment had been carried on during three months, and there could be no doubt that the tumours had disappeared. The question was, Had they been sarcoma? In regard to this, it is to be noted that, before the use of the Coley's fluid, the patient had for two months taken iodide of potassium in increasing doses up to 25 grains three times a day, without any effect whatever on the tumours, and that sections taken from the tumour over the sternum and also from over the clavicle had been examined by Dr. Jenner and Mr. Shattock, who had both assured Mr. Battle that the case was one of spindle-celled sarcoma, with some large cells. For all that it was held by some that this had not been a case of sarcoma, and it was asked that such cases should be "proved up to the hilt" before they were recorded as cases of cure of sarcoma by Coley's fluid. The point that at once arises is, how are we to prove such a case if such proofs as were offered by Mr. Battle are not to be accepted? It will be readily admitted that histology is not to be trusted when it is a matter of merely examining minute fragments of a tumour obtained by mechanical "fishing" arrangements, but when an honest slice is taken, and the results confirm those arrived at by clinical observation, it is difficult to see how in practice greater certainty can be arrived at, although we are free to admit that such evidence is not "proof," any more than is half the evidence on which we have to rely in the diagnosis of disease.

